# Supplementary material for: The co-crystal structure of Cbl-b and a small-molecule inhibitor reveals the mechanism of Cbl-b inhibition
Source: Commun Biol. 2023 Dec 16;6:1272. doi: 10.1038/s42003-023-05655-8 (PMC10725504; doi:10.1038/s42003-023-05655-8)
Supplement: Supplementary file 1 — Supplementary Information [file 42003_2023_5655_MOESM1_ESM.pdf]

SUPPLEMENTARY INFORMATION

The Co-crystal Structure of Cbl-b and a Small-Molecule Inhibitor Reveals the Mechanism of Cbl-b Inhibition

Serah W. Kimani[1,2], Sumera Perveen[1], Magdalena Szewezyk[1], Hong Zeng[1], Aiping Dong[1], Fengling Li[1], Pegah Ghiabi[1], Yanjun Li[1], Irene Chau[1], Cheryl H. Arrowsmith[1,2,3], Dalia Barsyte-Lovejoy[1,4], Vijayaratnam Santhakumar[1]*, Masoud Vedadi[4,5], Levon Halabelian[1,4]*

[1] Structural Genomics Consortium, University of Toronto, Toronto, Ontario, Canada
[2] Princess Margaret Cancer Center, University Health Network, Toronto, Ontario, Canada
[3] Department of Medical Biophysics, University of Toronto, Toronto, Ontario, Canada
[4]Department of Pharmacology and Toxicology, University of Toronto, Ontario, Canada
[5] Drug Discovery Program, Ontario Institute for Cancer Research, Toronto, Ontario, Canada

***To whom correspondence should be addressed.**

Levon Halabelian
Email: l.halabelian@utoronto.ca
Tel: +1 416-946-3876

Vijayaratnam Santhakumar

Email: santha.santhakumar@utoronto.ca
Tel: +1 416-978-7842

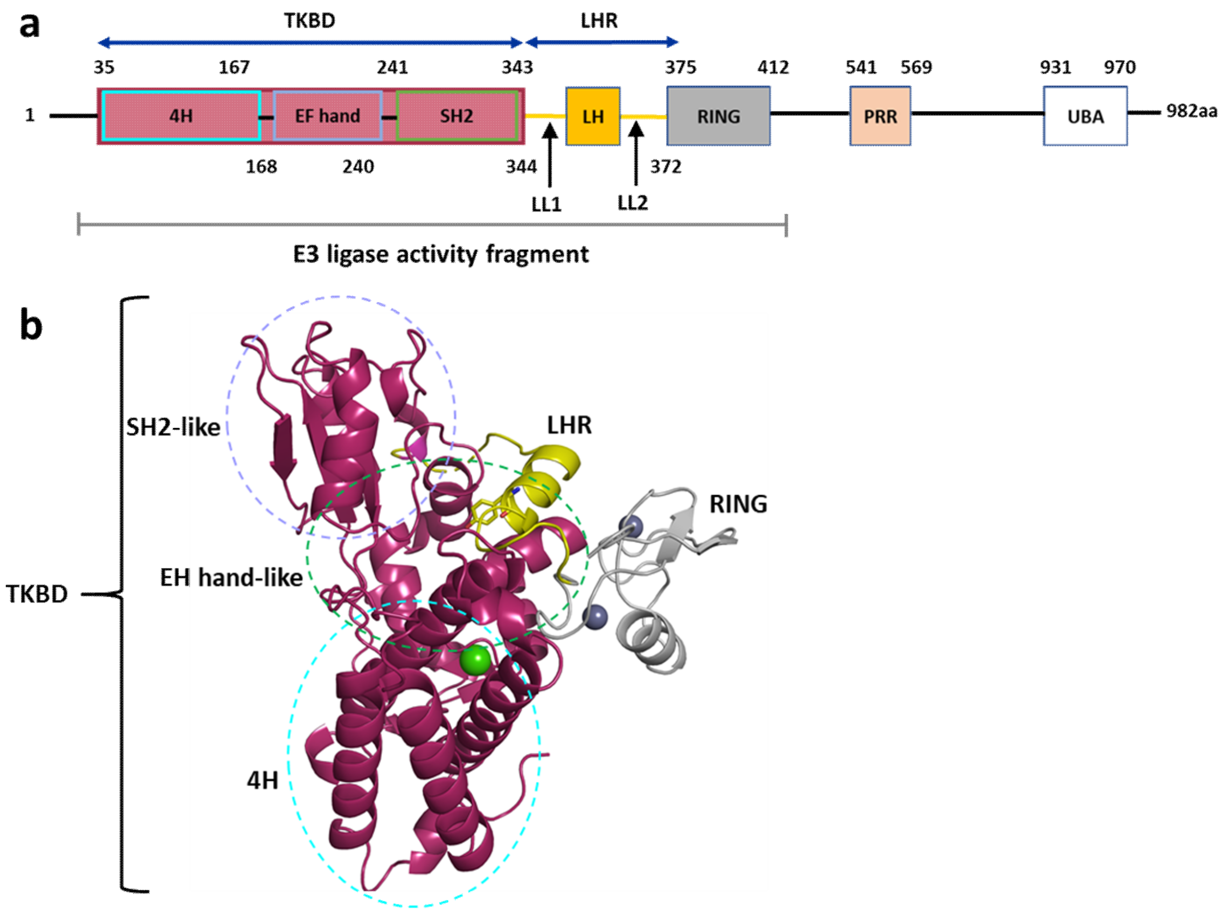

Cbl apo structure (PDB ID: 2Y1M)

Supplementary Figure 1: Cbl proteins domains. **a** A schematic of the full length Cbl-b protein. The N-terminal fragment that confers the Cbl-b E3 ligase activity is indicated and contains 3 domains including: the TKBD colored in magenta (made up of three subdomains, 4H, EF hand and SH2), the LHR shown in yellow and the RING finger domain in grey. **b** A cartoon representation of the c-Cbl apo protein structure (PDB ID: 2Y1M) showing the domains of the E3 ligase N-terminal fragment.

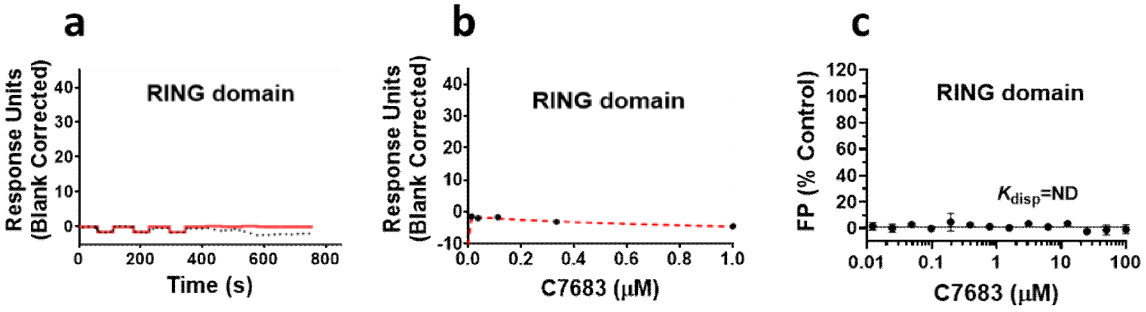

Supplementary Figure 2. Assessment of C7683 binding to Cbl-b RING domain by SPR and FP probe displacement. a-b Serially diluted C7683 was flown over immobilized RING domain protein (351-426aa). Representative sensorgram is shown in (solid red lines) with the kinetic fit (black dots) in **a**, and the steady-state response (black circles) with the steady state 1:1 binding model fitting (red dashed line) in **b**. **c** C7683 was tested for competing with the fluorescein-labeled probe (C7102) for binding to Cbl-b RING domain. C7683 didn't show any binding to the RING domain by SPR and FP. All experiments were performed in triplicates (n=3).

Supplementary Table 1. Summary of binding and peptide displacement assays. All values are the average ± standard deviation from experiments presented in figures 1 and 2 (n=3). SPR K_D values are from kinetic fitting.

**Target**	**Displacement** K_{disp} **(μM)**	**DSF** ΔT_m **(°C)**	**SPR** K_D **(nM)**
TKBD-LHR-RING	0.10 ± 0.02	10 ± 0.4	8 ± 4
Full-length	0.12 ± 0.02	12 ± 0.2	12 ± 6

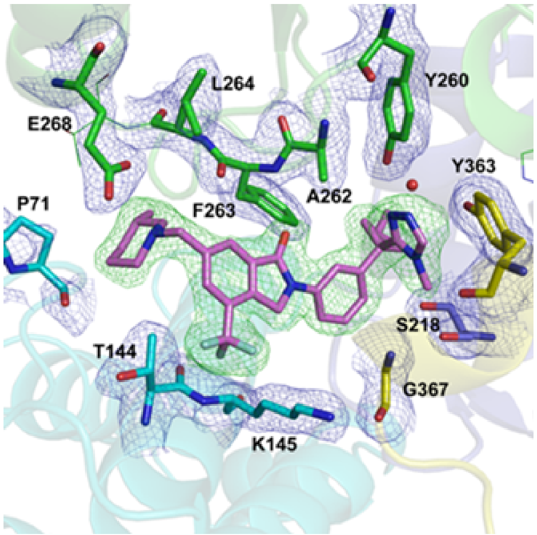

Supplementary Figure 3. Electron density map of C7683 bound to the TKBD subdomains and LHR of Cbl-b. The protein residues in the binding site are shown as sticks and are colored based on their respective domains as portrayed in figure 5A. A coordinating water molecule is rendered as a red sphere and the measured 2Fo-Fc electron density map around some of the highlighted residues in the vicinity of the compound is shown as blue mesh, contoured at 1.0σ level. C7683 electron density omit map (Fo-Fc) is shown as green mesh contoured at 3σ level and the C7683 compound is rendered as magenta sticks.

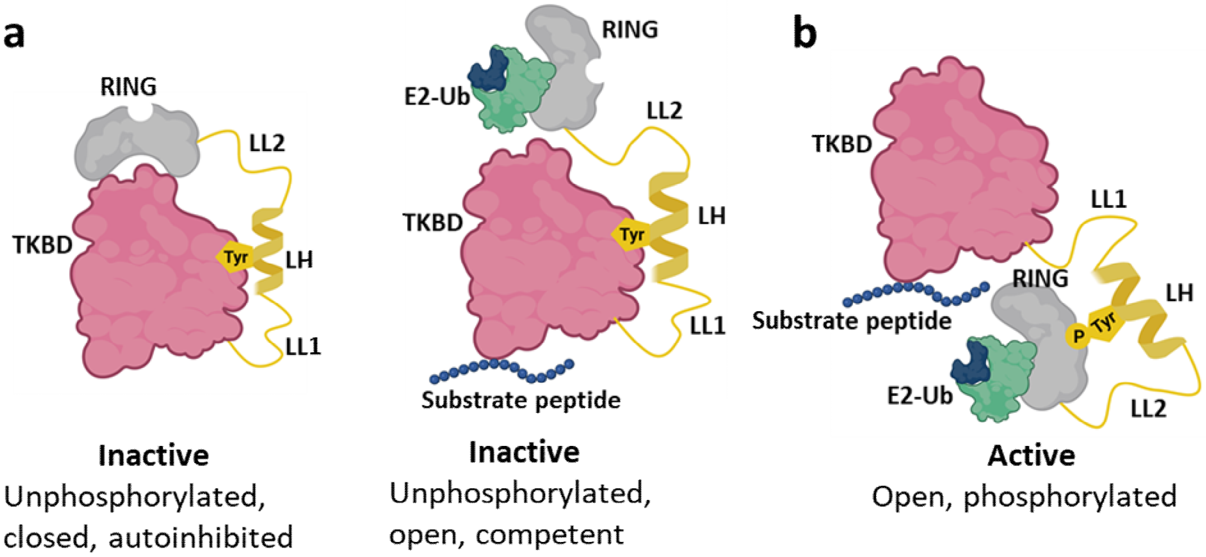

Supplementary Figure 4: A model of the LHR-mediated regulation of the Cbl proteins. a A model of the unphosphorylated inactive Cbl-b N-terminal fragment of Cbl-b, colored and labeled according to the schematic in Supplementary Figure 1. The LH is clamped onto the TKB domain, which restricts the movement of the RING finger domain to either closed and autoinhibited or open and competent of

binding to E2. The conserved tyrosine (Y363 in Cbl-b) is labeled as Tyr. **b** A model of the phosphorylated active Cbl-b, colored and labeled according to the schematic in panel A. Upon Y363 phosphorylation, the LH is released from the TKBD, and the RING domain is flipped around 180 degrees and moved adjacent to the substrate. The phosphorylated tyrosine is labeled (PTyr). Figure created with BioRender.com.
