# Supplementary material for: The co-crystal structure of Cbl-b and a small-molecule inhibitor reveals the mechanism of Cbl-b inhibition
Source: Commun Biol. 2023 Dec 16;6:1272. doi: 10.1038/s42003-023-05655-8 (PMC10725504; doi:10.1038/s42003-023-05655-8)
Supplement: Supplementary file 2 — Description of Additional Supplementary Files [file 42003_2023_5655_MOESM2_ESM.pdf]

Description of Additional Supplementary Files

File name: Supplementary Data 1

Description: The source data behind the graphs in figures 1, 2 and 3 in the paper and supplementary figure 2 in the supplementary information.
